# Factors affecting tracheostomy in critically ill paediatric patients in Japan: a data-based analysis

**DOI:** 10.1186/s12887-020-02144-3

**Published:** 2020-05-20

**Authors:** Tadashi Ishihara, Hiroshi Tanaka

**Affiliations:** Department of Emergency and Critical Care Medicine, Juntendo University, Urayasu Hospital, Urayasu-city, Chiba Japan

**Keywords:** Tracheostomy, Intensive care, Paediatric, Ventilation, Chronic condition, CPA

## Abstract

**Background:**

There has been an increasing number of children surviving with high medical needs, for whom tracheostomy and/or home ventilation is part of their chronic disease management. The purpose of this study was to describe the indications, epidemiology, frequency, and associated factors for tracheostomy in critically ill paediatric patients using the data available in the Japanese Registry of Paediatric Acute Care (JaRPAC).

**Methods:**

This multicentre epidemiologic study collected data concerning paediatric tracheostomy from the JaRPAC database. Patients were divided into two groups: those with or without tracheostomies when they were discharged from the Intensive Care Unit (ICU) or Paediatric Intensive Care Unit (PICU). Consecutive patients aged ≤16 years who did not undergo tracheostomy when admitted to ICU or PICU between April 2014 and March 2017 were included.

**Results:**

A total of 23 hospitals participated, involving 6199 paediatric patients registered in the JaRPAC database during the study period. Of the registered paediatric patients, 5769 (95%) patients were admitted to the ICUs or PICUs without tracheostomies. Among the patients, 181 patients (3.1%) had undergone tracheostomies. There were significant differences in chronic conditions (134, 74.0% versus 3096, 55.4%, *p* <  0.01), chromosomal anomalies (19, 10.5% versus 326, 5.8%, *p* <  0.01), urgent admission (151, 83.4% versus 3093, 55.4%, *p* <  0.01). More tracheostomies were performed on patients who were admitted for respiratory failure (61, 33.7% versus 926, 16.1%, *p* <  0.01) and for post-cardiac pulmonary arrest (CPA) resuscitation (40, 22.1% versus 71, 1.1%, *p* <  0.01).

**Conclusions:**

This is the first report to use a large-scale registry of critically ill paediatric patients in Japan to describe the interrelated factors of tracheostomies. Chronic conditions (especially for neuromuscular disease), chromosomal anomaly, admission due to respiratory failure, or treatment for post-CPA resuscitation all had the possibility to be risk factors for tracheostomy.

## Background

Previously, tracheostomy was performed primarily due to acute upper airway compromise secondary to infection [1–3]. Tracheostomy is a valuable procedure in children with severe respiratory compromise or upper airway obstruction. Recently, the clinical characteristics of children undergoing tracheostomy have changed, [1, 4–9] being performed most often in children who have an airway obstruction or those who require prolonged mechanical ventilation due to respiratory failure associated with chronic conditions, such as neuromuscular disease or bronchopulmonary dysfunction [8, 10]. The most common current indications of paediatric tracheostomy include prolonged ventilator dependence (as a consequence of prematurity and bronchopulmonary dysfunction) and upper airway obstruction (resulting either from craniofacial or structural abnormalities of the upper airway or hypotonia stemming from neurological or neuromuscular disturbance) [11]. Additionally, there has been an increasing number of children surviving with high medical needs for whom tracheostomy and/or home ventilation is part of their chronic disease management [4].

Determining whether paediatric patients are appropriate candidates for tracheostomy can be controversial, especially when the children have profound disabilities [12, 13]. Because there are currently no national or international recommendations regarding tracheostomy, the decision is currently based on clinical judgment [14]. In addition, little is known about the use of tracheostomy among paediatric patients requiring prolonged mechanical ventilation in the paediatric intensive care unit (PICU). There are also no published reports regarding the frequency of use, timing, and indication of tracheostomy of any cohort in Japan.

The purpose of this study was to describe the indications, epidemiology, frequency, and associated factors for tracheostomy in critical paediatric patients admitted to the intensive care unit (ICU) or PICU using the large amount of data available in the Japanese Registry of Paediatric Acute Care (JaRPAC).

## Methods

### Cohort selection

Consecutive patients aged ≤16 years who had no tracheostomy when admitted to ICUs or PICUs during the study period, between April 2014 and March 2017, were included in this study. Patients with tracheostomies before admittance to the ICU or PICU were excluded. Patients were not directly involved in the design of this study. All data were anonymized prior to their availability for this study by JaRPAC. This study was approved by the Institutional Review Board (30–025, in the Juntendo University Urayasu Hospital, Chiba, Japan), which waived the need for informed consent.

The JaRPAC is a multicentre clinical database of ICU and PICU paediatric patients that was founded by the Japanese Society for Emergency Medicine. It was initiated in April 2014, with the aim of evaluating critically ill paediatric patients and reducing their mortality rate. The JaRPAC database contains anonymized information regarding patient demographics, admissions, treatment, and outcomes, as well as scoring systems for severity and mortality [15]. Paediatric patients ≤16 years old in ICUs or PICUs are eligible for inclusion in this registry, and data are available on a per capita basis. The data were collected from admission until discharge from the ICU or PICU. The National Center for Child Health and Development is the primary institute managing this registry data, and hospitals that are affiliated with this institute are selected to participate in the registry. This includes twelve PICUs at children’s hospitals and eleven ICUs at critical care centres.

### Design

This was an epidemiologic study based on JaRPAC data. This study was subgroup analysis, by using JaRPAC data which was published by Ishihara T, et al. [16]. Data concerning patients who had not undergone tracheostomy when admitted to the ICU or PICU were extracted from the database. These patients were divided into two groups: those who received tracheostomies while in ICU or PICU (tracheostomy group) and patients without tracheostomies (no-tracheostomy group). Risk factors for tracheostomy were evaluated using the JaRPAC data. The cause of admission was divided into six categories: respiratory failure, circulatory failure, neurological dysfunction, post-operative care, tight observation, and recovery from cardiopulmonary arrest (CPA). The final diagnosis for each patient was registered and assigned as either an intrinsic or an extrinsic cause. Intrinsic disease was coded based on the International Classification of Diseases v. 10 (ICD-10) and categorized into one of ten groups (cardiovascular, respiratory, neuromuscular, gastrointestinal/hepato-biliary-pancreatic, haematologic/oncologic, renal, sepsis, metabolic/endocrinologic, allergic groups, and others) in order to ensure sufficient patients for analysis.

We used the Paediatric Index of Mortality 2 (PIM2) as a measure of severity for patients. The PIM2 score is calculated from various coefficients determined by Slater et al. [15] The values used to calculate PIM2 result from the first face-to-face contact between patients and physicians at ICUs or PICUs. Data for some factors were not obtained for all cases; these factors were not included in the PIM2 calculations in these cases. Patient survival was defined as discharge from an ICU or PICU.

Post-operative care admission was considered as elective admission. Admissions from general wards or transportation from other hospitals due to rapid deterioration or from the emergency department (ED) were considered urgent admissions. The duration of interventions performed in the ICU or PICU were compared between the groups. Interventions included continuous mechanical ventilation (CMV), central venous access catherization (CV), peripherally inserted central catheterization (PICC), and arterial line catherization (A-line).

We also evaluated complications, such as acute respiratory distress syndrome (ARDS) and ventilator associated pneumonitis (VAP). ARDS was defined by definition of Berlin criteria, and VAP was considered as a pneumonitis associated with a mechanical ventilation period lasting over 48 h [17, 18].

We defined chronic conditions according to Feudtner et al.’s definition, which states that a chronic condition ‘involves either several different organ systems or one organ system severely enough to require specialty paediatric care and probably some period of hospitalization in a tertiary care centre.’ [19] Chronic conditions were grouped into eight systems (cardiovascular, respiratory, neuromuscular, congenital/genetic abnormalities, gastrointestinal, renal, metabolic/endocrinologic and hematologic/immunologic), based on Feudtner’s complex chronic conditions. Children with multiple chronic conditions were counted multiple times, in each group corresponding to their conditions, for specific analysis, but were only counted once in the overall analysis. A clinically dominant chronic condition was defined as ‘the medical condition which carried the greatest morbidity for the child.’ [20]

### Statistical analysis

Data regarding age, length of PICU or ICU stay, PIM2, and length of interventions from JaRPAC were clearly skewed, so medians with interquartile ranges were used for numerical variables. Numerical variable differences between the two groups were compared using a Mann-Whitney U test. The chi-square test was used to compare sex distribution as well as frequencies of urgent admission, chronic conditions, chromosomal anomalies and complications. Data management and statistical analyses were undertaken using EZR software (Y Kaneda, Saitama, Japan). A *p*-value of < 0.05 was considered statistically significant.

## Results

**Fig. 1:**
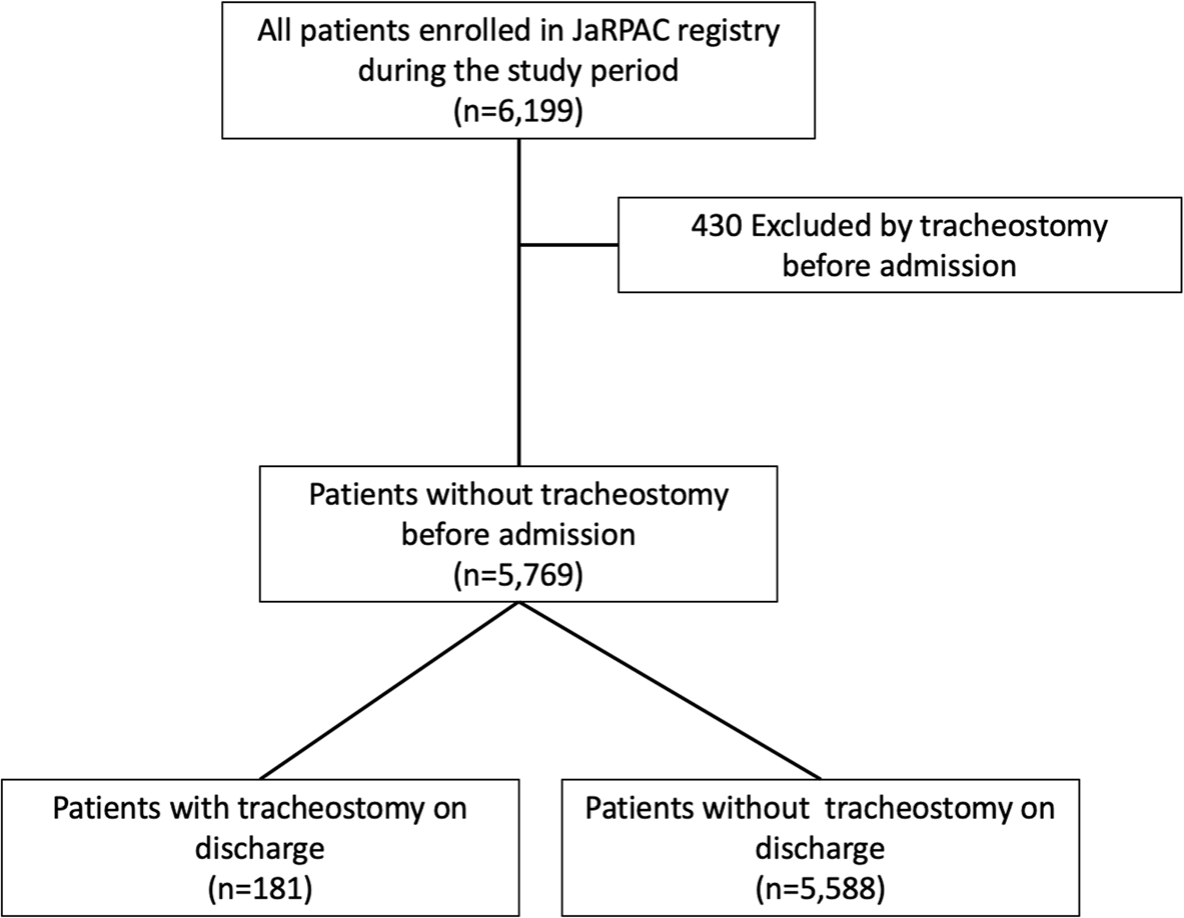
Study profile. JaRPAC: Japanese Registry of Paediatric Acute Care

A total of 23 hospitals contributed data that were used in the study, and 6199 paediatric patients were registered with JaRPAC during the study period. Of these patients, 5769 (95%) were admitted to the ICU or PICU without tracheostomies during the study period and were included in our study. There were 430 (5.0%) patients admitted with tracheostomies, who were excluded from our study. Among the 5769 enrolled patients, 181 patients (3.1%) belonged to the tracheostomy group and 5588 patients (96.9%) to the no-tracheostomy group (Fig. 1). The patients’ demographic characteristics are shown in Table 1. Forty-four patients in the tracheostomy group (24.3%) and 64 patients without tracheostomies (1.1%) died (*p* <  0.01). The median mortalities predicted by PIM2 were 11.1% (3.2–44.5) and 1.0% (0.3–2.4) for the tracheostomy and no-tracheostomy groups, respectively (*p* <  0.01). There were significant differences in the numbers of chronic conditions (*p* <  0.01), chromosomal anomalies (*p* <  0.01), and urgent admissions (*p* <  0.01) between the two groups.

**Table 1:** Characteristics of patients

Characteristics	total	no tracheostomy	tracheostomy	*p*-value
N	5769	5588	181	
Age (months)	25 (7–81)	24 (7–80)	36 (7–111)	0.06
Gender (male)	3037 (52.6)	2932 (52.5)	105 (58.0)	0.15
Length of PICU/ICU stay (days)	3 (2–6)	3 (2–6)	15 (4–26)	< 0.01
PIM2 (%)	1 (0.4–2.8)	1.0 (0.3–2.4)	11.1 (3.2–44.5)	< 0.01
Chronic condition	3230 (56.0)	3096 (55.4)	134 (74.0)	< 0.01
Chromosomal anomaly	345 (6.0)	326 (5.8)	19 (10.5)	< 0.01
Urgent admission	3244 (56.2)	3093 (55.4)	151 (83.4)	< 0.01
Mortality (%)	108 (1.9)	64 (1.1)	44 (24.3)	< 0.01

*PICU* Paediatric Intensive Care Unit, *ICU* Intensive Care Unit,

*PIM2* Paediatric Index of Mortality 2

Table 2 shows the causes for admission to ICUs and PICUs. Out of the 181 patients who received tracheostomies after admission, patients who were admitted for respiratory failure (61, 33.7% versus 926, 16.6%; *p* <  0.01) and for post-CPA resuscitation (40, 22.1% versus 71, 1.1%; *p* <  0.01) received significantly more tracheostomies.

**Table 2:** Admission reasons to ICU/PICU

Admission reasons	total	no tracheostomy	tracheostomy	*p*-value
N	5769	5588	181	
Respiratory failure (%)	987 (17.1)	926 (16.6)	61 (33.7)	< 0.01
Circulatory failure (%)	406 (7.0)	391 (7.0)	15 (8.3)	0.461
Neurological dysfunction (%)	996 (17.3)	968 (17.3)	28 (15.5)	0.617
Postoperative treatment (%)	2542 (44.1)	2515 (45.0)	27 (14.9)	< 0.01
Observation (%)	727 (12.6)	717 (12.8)	10 (5.5)	< 0.01
Treatment for post-CPA resuscitation (%)	111 (1.9)	71 (1.1)	40 (22.1)	< 0.01

*ICU* Intensive Care Unit, *PICU* Paediatric Intensive Care Unit,

*CPA* Cardiopulmonary arrest

Table 3 lists the therapies applied to and devices used by the patients. Significantly more patients in the tracheostomy group received CMV (181, 100% versus 2010, 36%; *p* <  0.01), CV line placement (118, 65.2% versus 1588, 28.4%; *p* <  0.01), A-line placement (156, 86.2% versus 2870, 51.4%; *p* <  0.01) and PICC placement (52, 29.3% versus 740, 13.2%; *p* <  0.01). Additionally, the duration of CMV (13 days [3–22.5] versus 3 days [2–6], *p* <  0.01), CV line (8 days [4–15.75] versus 4 days [3–7], *p* <  0.01), A-line (8.5 days [3–17] versus 3 days [2–6], *p* <  0.01) and PICC (10 days [5–24] versus 5 days [1–8], *p* <  0.01) were significantly longer in tracheostomy group than in the no tracheostomy group.

**Table 3:** Procedures at ICU/PICU

Procedures	total	no tracheostomy	tracheostomy	*p*-value
N	5769	5588	181	
CMV (%)	2191 (38)	2010 (36)	181 (100)	< 0.01
(days)	3 (2–6)	3 (2–6)	13 (3–22.5)	< 0.01
CV (%)	1706 (29)	1588 (28.4)	118 (65.2)	< 0.01
(days)	4 (3–7)	4 (3–7)	8 (4–15.75)	< 0.01
A line (%)	3026 (52)	2870 (51.4)	156 (86.2)	< 0.01
(days)	3 (2–6)	3 (2–6)	8.5 (3–17)	< 0.01
PICC (%)	793 (14)	740 (13.2)	53 (29.3)	< 0.01
(days)	5 (3–8)	5 (1–8)	10 (5–24)	< 0.01

*ICU* Intensive Care Unit, *PICU* Paediatric Intensive Care Unit,

*CMV* Continuous mechanical ventilation, *CV* Central venous, *A-line* Arterial line,

*PICC* Peripherally inserted central catheterization

Table 4 shows the categories of final diagnosis at ICUs and PICUs. The occurrence of extrinsic disease was significantly higher in the tracheostomy group (29, 16.0% versus 549, 9.8%; *p* <  0.01). Among intrinsic disease, respiratory disease was the leading diagnosis in the tracheostomy group, and its occurrence was significantly higher than in the no-tracheostomy group (72, 39.8% versus 1029, 18.4%; *p* <  0.01). Among the respiratory disease, pulmonary parenchyma disease, such as pneumonitis, was the leading cause of respiratory disease (Table 5).

**Table 4:** Categories of final diagnosis at ICU/PICU

Diagnosis	total	no tracheostomy	tracheostomy	*p*-value
N	5769	5588	181	
Neuromuscular disease (%)	1307 (22.7)	1279 (22.9)	28 (15.5)	0.0187
Respiratory disease (%)	1101 (19.1)	1029 (18.4)	72 (39.8)	< 0.01
Cardiovascular disease (%)	1062 (18.4)	1042 (18.6)	20 (11.0)	< 0.01
Gastrointestinal, Hepato-Bilary-Pancreatic disease (%)	609 (10.6)	602 (10.8)	7 (3.9)	< 0.01
Renal disease (%)	163 (2.8)	162 (2.9)	1 (0.6)	0.0647
Infectious disease (%)	157 (2.7)	153 (2.7)	4 (2.2)	1
Oncologic disease (%)	124 (2.1)	123 (2.2)	1 (0.6)	0.188
Metabolic/Endocrinologic disease (%)	86 (1.5)	84 (1.5)	2 (1.1)	1
Immunology disease	57 (1.0)	56 (1.0)	1 (1.0)	1
Other (%)	525 (9.1)	509 (9.1)	16 (8.8)	1

*ICU* Intensive Care Unit, *PICU* Paediatric Intensive Care Unit

**Table 5:** Type of respiratory disease

Type	total	no tracheostomy	tracheostomy	*p*-value
N	1101	1029	72	
Upper airway disease (%)	247 (22.4)	224 (21.8)	23 (31.9)	0.176
Lower airway disease (%)	274 (24.9)	261 (25.4)	13 (18.1)	
Pulmonary parenchyma disease (%)	458 (41.6)	431 (41.8)	27 (37.5)	
Others (%)	122 (11.1)	113 (11.0)	9 (12.5)	

Table 6 shows complications, with cases of ARDS and VAP being significantly higher in the tracheostomy than in the no-tracheostomy groups (25, 13.8% versus 59, 1.1%; *p* <  0.01; and 22, 12.2% versus 54, 1.0%; *p* <  0.01).

**Table 6:** Complications at ICU/PICU

Complications	total	no tracheostomy	tracheostomy	*p*-value
N	5769	5588	181	
ARDS (%)	84 (1.5)	59 (1.1)	25 (13.8)	< 0.01
VAP (%)	76 (1.3)	54 (1.0)	22 (12.2)	< 0.01

*ICU* Intensive Care Unit, *PICU* Paediatric Intensive Care Unit,

*ARDS* Acute respiratory distress syndrome, *VAP* Ventilator associated pneumonitis

Table 7 lists the chronic conditions of the patients. Fifty-one patients (34.7%) in the tracheostomy group and 923 patients (25%) in the no-tracheostomy group had neuromuscular disease as the most common chronic condition, but the difference was not significant.

**Table 7:** Chronic conditions

Chronic conditions	total	no tracheostomy	tracheostomy	*p*-value
N	3834	3687	147	
Neuromuscular disease (%)	974 (25.4)	923 (25)	51 (34.7)	0.012
Congenital/Genetic abnormality (%)	651 (17.0)	625 (17)	26 (17.7)	0.823
Cardiovascular disease (%)	598 (15.6)	578 (15.7)	20 (13.6)	0.563
Prematurity (%)	427 (11.1)	409 (11.1)	18 (12.2)	0.688
Respiratory disease (%)	362 (9.4)	351 (9.5)	11 (7.5)	0.474
Gastrointestinal, Hepato-bilary-pancreatic disease (%)	354 (9.2)	349 (9.5)	5 (3.4)	< 0.01
Hematological/Immunologic disease (%)	164 (4.3)	152 (4.1)	12 (8.2)	0.033
Metabolic/Endocrinologic disease (%)	154 (4.0)	151 (4.1)	3 (2)	0.248
Renal disease (%)	150 (3.9)	149 (4)	1 (0.7)	0.030

## Discussion

We found that patients admitted for respiratory failure or for recovery from CPA were the most likely to be given tracheostomies. Moreover, in the tracheostomy group, the duration of ICU or PICU stays were longer, and the PIM2-predicted mortality rate was higher. Chronic conditions, chromosomal anomalies and the rate of patient mortality were each significantly higher than they were for the control group. In addition, significantly more patients in the tracheostomy group had complications, such as ARDS or VAP, than those in the control group.

Our epidemiologic study of critical paediatric patients who had undergone tracheostomy provides details about the frequency of this intervention, as well as contrasting details about patients in ICUs and PICUs. In our epidemiologic study, 3.1% of the patients in ICUs and PICUs received tracheostomies, which is similar to the rate seen in other countries (1.8–6.6%). As such, our findings are consistent with other reports [21–23].

In our study, 74% of the paediatric patients in the tracheostomy group had chronic conditions. Edwards et al. also reported that the majority of patients in their tracheostomy group had chronic conditions that may have contributed to their airway compromise, and most of these patients had urgent admittance to ICUs or PICUs [21]. In a study in the UK, neuromuscular problems and chronic conditions were some of the factors cited as influencing the decision to perform a tracheostomy [22]. Berry et al. found that 48% of patients who received a tracheostomy at major children’s hospitals had a neurological impairment [24]. Some reports indicated that chronic conditions, such as neuromuscular problems or facial anomalies might be indications for tracheostomy [8, 14, 22]. As with other reports, the number and proportion of patients who had chronic conditions in our tracheostomy group was higher than that of our control group. In addition, the frequency of chronic conditions arising from neuromuscular disease was also significantly higher in the tracheostomy group than in the control group in our study.

It is important to highlight that, for many paediatric patients, tracheostomy intervention improves and prolongs life; furthermore, this intervention is sometimes temporary. For others, these dependencies are lifelong, but do not mitigate the patient’s other conditions; in these cases, tracheostomy intervention confers its own risk [25, 26]. As a result, questions about the eligibility of candidates for tracheostomy sometimes arise [13, 27–30].

The recommended timing or indication of tracheostomy for critical paediatric patients admitted to ICUs or PICUs for urgent care is not clear from available evidence [23]. As a result of changes to adult practice which have been driven by research data that are largely absent in the paediatric population, it is difficult to establish evidence-based indications for the paediatric population [22]. Prolonged intubation, ventilator dependence, and neurological or neuromuscular disorders can all be interrelated and difficult to separate. The complications, such as ARDS or VAP might be related to tracheostomy due to the longer mechanical ventilation. Having many classifications of indications can be helpful for the sake of specificity, but subclassifying the patient groups makes them much smaller, making meaningful comparison more difficult [11]. Consistent with a survey of Canadian paediatric intensivists, common indications of tracheostomy varied widely between institutes [31]. This means that there are no definitive guidelines. As a result, the indication and timing of tracheostomy depends on individual institutions or physicians. Koltai et al. reported that the long-term neurological status of children was the most consistent predictor of an ongoing tracheostomy requirement [32]. This is consistent with our study, in which the proportion of patients with neuromuscular disease and respiratory failure were significantly higher than in the control group.

Our study has several limitations. First, we conducted a retrospective analysis; therefore, only associations among the available data could be described. Second, there were no data relevant to definitive indications or timing of tracheostomy available. Additionally, there was no data available about the decision-making process, role of parents or caregivers, palliative care, and do-not attempt resuscitation order. Third, the data about tracheostomy were evaluated by univariate analysis; hence, careful interpretation of these results is needed. Finally, the JaRPAC might have a selection bias if disproportionately more academically focused or resource-rich ICUs and PICUs joined the database. Whether this is the case is uncertain since this registry database does not provide institutional characteristics and therapeutic levels.

## Conclusions

This study used a large-scale registry of critically ill paediatric patients in Japan to describe the interrelated factors of patients who had undergone tracheostomies in ICUs or PICUs. Chronic conditions (especially for neuromuscular disease), chromosomal anomaly, admission due to respiratory failure (especially for pulmonary parenchyma disease), or treatment for post-CPA resuscitation all had the possibility to be risk factors for tracheostomy.

Further prospective studies are needed to reveal the risk factors of tracheostomy for critical ill patients.

## Data Availability

The datasets analysed during the current study are not publicly are not publicly available due to contain each patient’s characteristics of participated hospital, but are available from the corresponding author on reasonable request.

## Abbreviations

JaRPAC: Japanese registry of paediatric acute care
ICU: Intensive care unit
PICU: Paediatric intensive care unit
CPA: Cardiac pulmonary arrest
ICD-10: International classification of diseases v. 10
PIM2: Paediatric index of mortality 2
ED: Emergency department
CMV: Continuous mechanical ventilation
CV: Central venous access catherization
PIPC: Peripherally inserted central catheterization
A-line: Arterial line catherization
ARDS: Acute respiratory distress syndrome
VAP: Ventilator associated pneumonitis
